# Local gradient analysis of human brain function using the Vogt-Bailey Index

**DOI:** 10.1007/s00429-023-02751-7

**Published:** 2024-01-31

**Authors:** Christine Farrugia, Paola Galdi, Irati Arenzana Irazu, Kenneth Scerri, Claude J. Bajada

**Affiliations:** 1 Faculty of Engineering, L-Università ta’ Malta, Msida, Malta; 2 University of Malta Magnetic Resonance Imaging Platform (UMRI), L-Università ta’ Malta, Msida, Malta; 3 Centre for Clinical Brain Sciences, The University of Edinburgh, Edinburgh, UK; 4 School of Informatics, The University of Edinburgh, Edinburgh, UK; 5 Faculty of Engineering, Mondragon Unibertsitatea, Mondragón, Spain; 6 Faculty of Medicine and Surgery, L-Università ta’ Malta, Msida, Malta

**Keywords:** Cortical organization, Functional connectivity, Vogt-Bailey index, Regional homogeneity

## Abstract

In this work, we take a closer look at the Vogt-Bailey (VB) index, proposed in Bajada et al. (NeuroImage 221:117140, 2020) as a tool for studying local functional homogeneity in the human cortex. We interpret the VB index in terms of the minimum ratio cut, a scaled cut-set weight that indicates whether a network can easily be disconnected into two parts having a comparable number of nodes. In our case, the nodes of the network consist of a brain vertex/voxel and its neighbours, and a given edge is weighted according to the affinity of the nodes it connects (as reflected by the modified Pearson correlation between their fMRI time series). Consequently, the minimum ratio cut quantifies the degree of small-scale similarity in brain activity: the greater the similarity, the ‘heavier’ the edges and the more difficult it is to disconnect the network, hence the higher the value of the minimum ratio cut. We compare the performance of the VB index with that of the Regional Homogeneity (ReHo) algorithm, commonly used to assess whether voxels in close proximity have synchronised fMRI signals, and find that the VB index is uniquely placed to detect sharp changes in the (local) functional organization of the human cortex.

**Supplementary Information:**

The online version contains supplementary material available at 10.1007/s00429-023-02751-7.

## Introduction

Since the emergence of neuroscience as a distinct discipline, there has been increasing interest in understanding the organizational principles of the cerebral cortex. The degree to which the cortex is parcellated into separate regions has been strongly debated over the years. Oskar and Cécile Vogt, well known for their myeloarchitectonic cortical maps, counselled sharp, ‘hairline’ boundaries [(Nieuwenhuys 2013) and references therein]. The cytoarchitectonic parcellation described by Korbinian Brodmann (Brodmann 1909) also divides the cortex into different areas, although Brodmann himself pointed out that in some cases, the boundaries are not sharp and changes occur gradually (Brodmann 1909). On the other hand, Percival Bailey and Gerhardt von Bonin advocated that the isocortex is characterised by a high degree of homogeneity (Bailey and von Bonin 1951). This raises the question: is it really appropriate to parcellate the cortex into distinct regions?

The rapid advance of new technologies and introduction of techniques such as magnetic resonance imaging (MRI), coupled with developments in the fields of graph theory and network analysis, gave new impetus to the study of cortical organization. The aim of applying graph theory to neural data is to investigate the emerging connectivity patterns, which reveal how different brain areas are related to each other structurally or functionally (Farahani et al. 2019). The use of networks in neuroscience can provide important insight into human cognition and behaviour (Kriegeskorte and Douglas 2018; Sporns 2014), and further our understanding of how the brain changes with age (Betzel et al. 2014; Cao et al. 2014; Grayson and Fair 2017), how it adapts itself to various cognitive demands (Bassett et al. 2011), and how intelligence and intellectual abilities are related to functional connectivity (van den Heuvel et al. 2009; Wu et al. 2013). Functional networks have also been widely studied in the context of neurological and psychiatric disorders, such as the degenerative dementias (Pievani et al. 2011), epilepsy (Výtvarová et al. 2017), multiple sclerosis (Schoonheim et al. 2014), schizophrenia (Kambeitz et al. 2016), depression (Zhi et al. 2018), and autism spectrum disorder (Soma et al. 2021). In recent years, the concept of *gradients* (or spatial transitions) in brain organization (Bernhardt et al. 2022) has become an especially popular topic. These gradients are usually used to infer how the ‘activation hubs’ for a certain class of functions are distributed within the brain.

In the majority of cases, neuroscientific works consider connections between different brain regions (or ‘parcels’). However, methods from graph theory can also quantify the affinity between two or more adjacent vertices/voxels. This property makes them a good tool to study the local disruptions in brain function implicated in certain disorders/diseases (Kozhemiako et al. 2020; Wei et al. 2018; Keown et al. 2013). The searchlight Vogt-Bailey (VB) algorithm developed in Bajada et al. (2020) determines functional connectivity on a per-vertex level by constructing, for each vertex on the surface of the brain, a graph consisting of the original vertex and its neighbours. The vertices are treated as the nodes of the graph, while the (modified) Pearson correlation between their functional MRI (fMRI) time series is used to assign weights to the connecting edges. The algebraic connectivity, equivalent to the second smallest eigenvalue of the graph Laplacian, indicates how easy or difficult it is to disconnect the graph,^1^ and thus serves to gauge the degree of homogeneity around the original vertex. The VB index is defined as a scaled version of the algebraic connectivity. It can additionally be adapted to serve as a metric for full brain or region-of-interest analysis, depending on the size of the neighbourhood that is provided as input. The VB toolbox is available at https://github.com/VBIndex.

The searchlight VB index has been used (albeit in voxel space) to explore the organization of axonal fibres by providing a measure of the correlation between the connection probabilities of neighbouring voxels (Lee and Park 2022). The authors modelled the connection probability of a given voxel as a data series consisting of 360 elements, with each element reflecting the probability of tract linkage between the voxel in question and one of 360 target cortical regions (Lee and Park 2022). In another study, the VB index was employed to probe the functional organization of the rodent hippocampus and indicated a sharp change in connectivity (Gunnarsdóttir et al. 2022).

The mathematical basis of the VB index is well established. In fact, a number of research articles (such as Benkarim et al. 2021; Margulies et al. 2016; Jackson et al. 2018; Glomb et al. 2021) use the same framework to describe how neural gradients are mapped and how inter-areal boundaries can be identified. The searchlight^2^ VB index differs in that it shifts the focus from the global level to the local—it is calculated by constructing networks on a small scale (one per vertex), rather than across a region of interest or the entire brain.

One shortcoming of local analysis is the artificial enhancement of correlations that may result from volume-to-surface mapping, especially in the vicinity of narrow sulci and gyri (Ciantar et al. 2022). This gets more pronounced when surface resolution is increased with respect to voxel data resolution, since more surface vertices sample the same voxels. To mitigate the issue, another VB index approach was developed—the hybrid searchlight algorithm. This estimates the algebraic connectivity in volumetric space and maps the results to the original surface vertices (Ciantar et al. 2022). A modified version was used with diffusion data to study the impact of preterm birth on the homogeneity of tissue microstructure in the neonatal cortex (Galdi et al. 2022). Indeed, the methodology underlying the VB algorithm can be adapted to different modalities of data. In this article, we focus on its application to functional MRI data, as in its current form, the VB toolbox is intended for such a purpose.

The work presented here complements the original article (Bajada et al. 2020) by unravelling the details of the mathematical framework of the VB index. In the “Methods” section, we first take a look at some principles from graph theory, then proceed to an interpretation of the VB index as a cut-set weight corresponding to a cut that partitions the graph into two while attempting to minimise any imbalance in cluster size. In this way, we lay the theoretical groundwork for the VB index, which motivates our investigation of the eigenvalue problem that is central to the algorithm (Bajada et al. 2020) and in particular, of the difference between the results obtained with the generalised and standard versions of said problem. We test the two on synthetic data and show that the latter is advantageous, and also derive a scaling factor that is in line with our adoption of the standard eigenvalue problem. This marks a significant deviation from the approach taken by Bajada et al. (2020) and an improvement of the searchlight functionality of the VB index.

The “Results” section is dedicated to experimental validation, including comparison with the Regional Homogeneity (ReHo) metric, which measures the degree of synchronisation in the time series of neighbouring voxels (Zang et al. 2004). ReHo is often used in combination with functional connectivity analysis to study whether certain disorders or diseases are associated with changes in local brain activity. Indeed, this strategy has been utilised in the case of hepatitis B virus-related cirrhosis patients with or without minimal hepatic encephalopathy (Sun et al. 2018), as well as for patients with bipolar II disorder (Xu et al. 2019) or attenuated psychosis syndrome (Long et al. 2018). Additionally, abnormal ReHo values have been detected in subjects with early- or late-onset Parkinson’s disease (Yue et al. 2020) and acute or remitting multiple sclerosis (Zhu et al. 2020), among others. ReHo maps can potentially serve as a non-invasive prognostic tool for cirrhotic patients with overt hepatic encephalopathy (Lin et al. 2015), and have a high diagnostic accuracy for congenital blindness (Hu et al. 2022). Real-time fMRI neurofeedback and associated brain function self-regulation were found to impact the ReHo scores of brain regions involved in the processing of emotions (Yang et al. 2018). In another study investigating the test–retest reliability of ReHo (Zuo et al. 2013), the authors found that this could be improved by employing a fast imaging sequence, using nuisance correction but no spatial smoothing in the preprocessing stage, and by carrying out the analysis on the surface of the brain (in a vertex-wise manner, rather than the voxel-wise implementation of Zang et al. 2004).

The VB index differs from ReHo in two significant ways: first, it takes into account the values of the data points in the fMRI time series, not just their rankings. Second, our framework admits a degree of flexibility—in that the similarity metric, which in our case is a modified Pearson correlation coefficient, can be replaced without compromising the inherent attributes of the VB index. Given the recent surge in the community’s interest in local functional connectivity, this paper undertakes to compare the performance of the ReHo and VB index algorithms, and to determine whether the latter can take us a step further in gauging the local homogeneity of brain activity and what it means.

## Methods

### Spectral graph theory: optimisation as an eigenvalue problem

#### Preliminaries

Let $G=(V,E)$ be an undirected, weighted graph with vertex set *V* of size *n* and edge set $E=\{(v_i,v_j)\in V\times V,\,v_i\ne v_j\}$. *G* has no loops (i.e., no edges starting and terminating at the same vertex), and direct connections between any two vertices are limited to at most one edge. To simplify notation, we shall refer to the edge joining vertices $v_i$ and $v_j$, $(v_i,v_j)$, as $e_{ij}$. The weight associated with $e_{ij}$ will be denoted by $w_{ij}$ and is a number in the interval [0, 1].

To determine the best way of partitioning *V* into two disjoint clusters, we introduce the cost function *U*:

1 $$\begin{aligned} U = \sum _{(i,j)|e_{ij}\in E} w_{ij}(x_i-x_j)^2. \end{aligned}$$

The variables $x_i$ and $x_j$ are the positions of the vertices $v_i$ and $v_j$, respectively; we emphasise, however, that they do *not* refer to the positions that the vertices have in 2D Euclidean space (such as in Fig. 2), but rather to coordinates in 1D space. In other words, we map the vertices to a line, much like the beads in one row of an abacus frame, and let the mathematics adjust the vertices (‘beads’) until the separations between them are optimal—in the sense that they make *U* as small as possible. The optimization algorithm looks for a trade-off between the weights of the edges and the separation of the respective vertices. Since we want to minimise *U*, an edge with substantial weight will tend to be shorter, so that the small value of $(x_i-x_j)^2$ compensates for the large $w_{ij}$; consequently, in such cases, $x_i$ and $x_j$ are usually either both positive or both negative. On the other hand, edges with a relatively small weight can afford to be longer (one might argue that keeping them short would be even better, as it would decrease the cost function further; however, it must be remembered that moving one vertex with respect to another also moves it relative to the remaining vertices, so the question is how to balance edge weights and vertex separations). The end result is that vertices which are strongly connected to each other will usually cluster on one side of the zero reference point when mapped to a line, while vertices sharing a weak connection are further apart and often tend to be positioned on different sides of the zero reference point.

Next, we construct a vector $\mathbf {{x}}$ consisting of as many components as there are vertices, with each component $x_i$ being the position of the corresponding vertex $v_i$ on the line. Since Eq. (1) only constrains the difference between the elements of $\mathbf {{x}}$, and not the elements per se, it could trivially be satisfied by setting $x_i=x_j$ for all vertices $v_i$ and $v_j$. However, this would hardly constitute a useful solution, as what we are looking for is a graph whose structure can give us information about the way the distinct vertices are related. Collapsing the graph to a single point would cause it to lose any structure and the distinctiveness of its vertices. We therefore build on the example of Von Luxburg (2007) and impose the condition

2 $$\begin{aligned} \textbf{x}\cdot \textbf{1} = \sum _{i=1}^{n} x_i = 0~;~~~{\sum _{i=1}^{n} |x_i| \ne 0,} \end{aligned}$$

where $\textbf{1}$ is the all-ones vector of size *n* and $|x_i|$ the absolute value of $x_i$. Equation (2) implies that the components of $\mathbf {{x}}$ cannot be all positive or all negative, and is essentially an attempt to get well-balanced clusters distributed on each side of the zero reference point.

Let us now take a short detour to define some terminology related to graph theory, starting from the (weighted) *affinity matrix* $\textbf{A}$, whose elements are given by

3 $$\begin{aligned} a_{ij}={\left\{ \begin{array}{ll} w_{ij}\quad \text {if}~i\ne j;\\ 0~\qquad \text {if}~i=j. \end{array}\right. } \end{aligned}$$

Since the graph is assumed to be undirected, it follows that $w_{ij}=w_{ji}$, and hence $\textbf{A}$ is symmetric. In our case, $w_{ij}$ will be calculated as the quantity

4 $$\begin{aligned} w_{ij}=1-\left\{ \arccos {[\text {corr}(\textbf{s}_i,\textbf{s}_j)]} \times \frac{180}{\pi }\times \frac{1}{90}\right\} , \end{aligned}$$

$\text {corr}(\textbf{s}_i,\textbf{s}_j)$ being the sample Pearson correlation between the time series $\textbf{s}_i$ and $\textbf{s}_j$ associated with the vertices $v_i$ and $v_j$, respectively. The (sample) Pearson correlation is equivalent to the cosine similarity for the mean-centred versions of the time series: $\text {corr}(\textbf{s}_i,\textbf{s}_j) = \cos {(\textbf{s}_i-\bar{\textbf{s}}_i,\textbf{s}_j-\bar{\textbf{s}}_j)}$. However, the sinusoidal nature of the cosine function means that it does not map the range of angles between $0^\circ$ and $90^\circ$ linearly to the interval [0, 1]. For instance, two vectors angled at $45^\circ$ would have a cosine similarity of 0.7 rather than 0.5. To recover this linearity, we retrieve the angles by taking the inverse cosine and (after converting to degrees) divide by $90^\circ$ to rescale [0, 90] to [0, 1]. Finally, we subtract the result from unity, so that identical signals (which can be treated as parallel vectors, giving $\arccos {[\cos {(0^\circ )]}=0}$) are assigned a weight of 1, not 0, whereas signals that are completely uncorrelated yield $w_{ij}=0$. We emphasise that the general principle behind the VB index is independent of the choice of similarity metric.

Another array we shall be using is the *degree matrix* $\textbf{D}$. This is a diagonal matrix which may be constructed from $\textbf{A}$ by summing its entries either row-wise or column-wise. More specifically,

5 $$\begin{aligned} d_{ij}={\left\{ \begin{array}{ll} 0~\text {if}~i\ne j~;\\ \sum _{k=1}^n a_{ik}=\sum _{\begin{array}{c} k=1\\ k\ne i \end{array}}^n w_{ik}~\text {if}~i=j. \end{array}\right. } \end{aligned}$$

The equality in the last line follows from Eq. 3, and the quantity $\sum _{k=1}^n w_{ik}$ $(k\ne i)$ represents a sum over the weights of the edges joining $v_i$ to the remaining $n-1$ vertices of the graph (this does not mean the graph is complete; we treat absent edges as edges having a weight of zero). The *i*th element along the principal diagonal of $\textbf{D}$, $d_{ii}$, is known as the *degree* of the vertex $v_i$.

The *graph Laplacian* $\textbf{L}$ is defined as

6 $$\begin{aligned} \textbf{L}=\textbf{D}-\textbf{A} \end{aligned}$$

and is a symmetric, positive semi-definite matrix. The spectrum of eigenvalues of the Laplacian can be determined by solving the eigenvalue equation

7 $$\begin{aligned} \textbf{L}\mathbf {{u}} = \lambda \mathbf {{u}}. \end{aligned}$$

Vectors $\mathbf {{u}}$ (excluding $\mathbf {{0}}$, which represents the trivial solution) and values of $\lambda$ that satisfy this equation are called *eigenvectors* and *eigenvalues*, respectively; each eigenvalue is paired with at least one eigenvector. The graph Laplacian has the smallest eigenvalue equal to 0 and the all-ones vector $\mathbf {{1}}$ as the corresponding eigenvector. The second smallest eigenvalue is known as the *algebraic connectivity* (Fiedler 1973) and is the quantity we will be using to construct the VB Index.

#### Minimising the Rayleigh quotient

It can be shown that the quadratic cost function of Eq. (1) may be expressed in terms of the Laplacian via the relation^3^

8 $$\begin{aligned} \frac{1}{2}\sum _{i,j=1}^n w_{ij}(x_i-x_j)^2= \textbf{x}^\text {T}\textbf{L}\textbf{x}. \end{aligned}$$

Consequently, the problem of partitioning the graph in the manner outlined above can mathematically be formulated [using Eq. (1)] as the requisite to minimise the Rayleigh quotient

9 $$\begin{aligned} U(\textbf{x}) = \frac{\textbf{x}^\text {T}\textbf{L}\textbf{x}}{\textbf{x}^\text {T}\textbf{x}}~;~~~\textbf{x}\ne \textbf{0}, \end{aligned}$$

subject to the condition^4^$\textbf{x}\cdot \textbf{1}=0$ [Eq. (2)]. We have introduced the magnitude of $\mathbf {{x}}$ in the denominator to avoid getting solutions which optimise the cost function by making the components of $\mathbf {{x}}$ arbitrarily small.

The vector $\mathbf {{x}}$ which minimises $U(\mathbf {{x}})$ is none other than the Fiedler vector, the eigenvector associated with the second smallest eigenvalue of the Laplacian matrix. This can be proved as follows (refer to Stanković et al. (2019) and to *Introduction to spectral graph theory* by A. J. Ganesh [https://people.maths.bris.ac.uk/~maajg/teaching/complexnets/laplacians.pdf]):

Let $\textbf{L}$ have eigenvalues $0=\lambda _1<\lambda _2\le \dots \le \lambda _n$ with corresponding orthonormal eigenvectors^5^$\mathbf {{u}}_1, \mathbf {{u}}_2, \dots \mathbf {{u}}_n$. As the eigenvectors provide an orthonormal basis, any vector $\mathbf {{x}}$ may be expressed in the form

10 $$\begin{aligned} \mathbf {{x}} = \sum _{i=1}^n c_i\mathbf {{u}}_i, \end{aligned}$$

where $c_i=\mathbf {{x}}\cdot \mathbf {{u}}_i=\mathbf {{x}}^\text {T}\mathbf {{u}}_i$. Hence, we get that

11 $$\begin{aligned} \mathbf {{x}}^\text {T}\textbf{L}\mathbf {{x}}&= \bigg (\sum _{j=1}^n c_j\mathbf {{u}}_j\bigg )^{\!\!\text {T}}\textbf{L}\bigg (\sum _{i=1}^n c_i \mathbf {{u}}_i\bigg )\nonumber \\&= \bigg (\sum _{j=1}^n c_j\mathbf {{u}}_j\bigg )^{\!\!\text {T}}\sum _{i=1}^n c_i\lambda _i\mathbf {{u}}_i\nonumber \\&= \sum _{j=1}^n\sum _{i=1}^n c_j^{} c_i^{}\lambda _i^{}\,\mathbf {{u}}_j^\text {T}\mathbf {{u}}_i^{}=\sum _{i=1}^n c_i^2\lambda _i^{}, \end{aligned}$$

since the eigenvectors are orthonormal (meaning that $\mathbf {{u}}_j^\text {T}\mathbf {{u}}_i^{}=1$ if $i=j$, and 0 otherwise). In similar fashion,

12 $$\begin{aligned} \mathbf {{x}}^\text {T}\mathbf {{x}} = \bigg (\sum _{j=1}^n c_j\mathbf {{u}}_j\bigg )^{\!\!\text {T}}\bigg (\sum _{i=1}^n c_i\mathbf {{u}}_i\bigg ) = \sum _{i=1}^n c_i^2. \end{aligned}$$

Suppose, now, that $\mathbf {{x}}$ is orthogonal to $\mathbf {{1}}$, i.e., $\mathbf {{x}}\cdot \mathbf {{1}}=\mathbf {{x}}^\text {T}\mathbf {{1}}=0$. Given that $\mathbf {{u}}_1=\mathbf {{1}}/\sqrt{n}$ , and that the component of $\mathbf {{x}}$ along $\mathbf {{u}}_1$, $c_1$, is obtained by taking the dot product of $\mathbf {{x}}$ with $\mathbf {{u}}_1$, it follows that $c_1 = \mathbf {{x}}\cdot \mathbf {{u}}_1 = (\mathbf {{x}}\cdot \mathbf {{1}})/\sqrt{n}=0$. Consequently, we can drop $c_1$ from the sums in Eqs. (11) and (12), and write

13 $$\begin{aligned} \frac{\mathbf {{x}}^\text {T}\textbf{L}\mathbf {{x}}}{\mathbf {{x}}^\text {T}\mathbf {{x}}}=\frac{\sum _{i=2}^n c_i^2\lambda _i^{}}{\sum _{j=2}^n c_j^2}\ge \frac{\sum _{i=2}^n c_i^2\lambda _2^{}}{\sum _{j=2}^n c_j^2}=\lambda _2. \end{aligned}$$

If $$\mathbf {{x}}=\mathbf {{u}}_2,$$

14 $$\begin{aligned} \frac{\mathbf {{x}}^\text {T}\textbf{L}\mathbf {{x}}}{\mathbf {{x}}^\text {T}\mathbf {{x}}} =\frac{\mathbf {{u}}_2^\text {T}\textbf{L}\mathbf {{u}}_2^{}}{\mathbf {{u}}_2^\text {T}\mathbf {{u}}_2^{}} =\frac{\mathbf {{u}}_2^\text {T}\lambda _2^{}\mathbf {{u}}_2^{}}{\mathbf {{u}}_2^\text {T}\mathbf {{u}}_2}=\lambda _2. \end{aligned}$$

In conclusion, then, any vector $\mathbf {{x}}$ orthogonal to $\mathbf {{1}}$ satisfies

15 $$\begin{aligned} \frac{\mathbf {{x}}^\text {T}\textbf{L}\mathbf {{x}}}{\mathbf {{x}}^\text {T}\mathbf {{x}}}\ge \lambda _2, \end{aligned}$$

with equality attained when $\mathbf {{x}}$ is set to $\mathbf {{u}}_2$, the Fiedler vector. To recap, we have thus far:

- Considered a general vector $\mathbf {{x}}$ whose elements correspond to the positions of the vertices in 1D space;
- Shown that the partitioning of the graph is optimized [with respect to Eq. (1)] if $\mathbf {{x}}$ is the Fiedler vector.

In other words, if we would like to separate the graph into two balanced clusters in a way that minimises the cost function given by Eq. (9), the vertex $v_i$ should be mapped to the coordinate $x_i$ in 1D space given by the *i*th component of the Fiedler vector. Vertices that end up in close proximity in this 1D setting can be grouped together when the original graph is partitioned.

#### The generalised vs standard eigenvalue problem

Let us go back to the equation whence the Fiedler vector originated; namely, the standard eigenvalue equation:

16 $$\begin{aligned} \textbf{L}\mathbf {{x}} = \lambda \mathbf {{x}}. \end{aligned}$$

Outlying vertices are likely to have significant effect on the clustering, but we can reduce their influence using the *generalised eigenvalue problem* in place of the standard one:

17 $$\begin{aligned} \textbf{L}\mathbf {{x}} = \lambda \textbf{D}\mathbf {{x}}, \end{aligned}$$

$\textbf{D}$ being the degree matrix introduced in Eq. (5). If we now multiply both sides of Eq. (17) by the inverse of the degree matrix, $\textbf{D}^{-1}$, we get the relation

18 $$\begin{aligned} \textbf{D}^{-1}\textbf{L}\mathbf {{x}} = \lambda \mathbf {{x}}. \end{aligned}$$

Using the definitions of the degree matrix and the Laplacian from Eqs. (5) and (6), respectively, it is straightforward to show that the matrix product $\textbf{D}^{-1}\textbf{L}$ (which is known as the *random walk normalised Laplacian* and will be denoted by $\textbf{L}_\text {RW}$) takes the form

19 $$\begin{aligned} \textbf{L}_\text {RW} = \begin{pmatrix} 1 &{} -w_{12}/d_{11} &{} \dots &{} -w_{1n}/d_{11}\\ -w_{21}/d_{22} &{} 1 &{} \dots &{} -w_{2n}/d_{22}\\ \vdots &{} \vdots &{} \ddots &{} \vdots \\ -w_{n1}/d_{nn} &{} -w_{n2}/d_{nn} &{} \dots &{} 1\\ \end{pmatrix}, \end{aligned}$$

with $d_{ii} = \sum _{k=1}^n w_{ik}~(k\ne i)$ and $w_{ij}=w_{ji}$. Equation (17), then, becomes equivalent to $\textbf{L}_{\text {RW}}\mathbf {{x}} = \lambda \mathbf {{x}}$, the standard eigenvalue equation for the Laplacian $\textbf{L}_{\text {RW}}$ of a graph whose vertices all have degree 1. This new graph can be thought of as a derivative of the original, obtained by adjusting weights: if a vertex is connected to edges with large weights or if it has many neighbours—in other words, if it is strongly connected—the weights of the incident edges are scaled down. The opposite happens for a weakly connected vertex. Note that the random walk normalised Laplacian is not symmetric—indicating that the new graph is directed, i.e., the weights depend on the direction in which the edges are traversed.^6^

The weight redistribution described above can be made more intuitive by analogy with people’s following on social media profiles, which may be regarded as a measure of their ‘degree of friendship’. Someone mainly interested in having a large following would likely accumulate a significant number of remote acquaintances among their connections. On the other hand, people who only connect with close friends would have a smaller following. In the former case, the ‘degree of friendship’ would have to be scaled down, because a good proportion of connections would not represent meaningful friendships, while for the latter the percentage of followers who are intimate friends would be larger, and hence the degree of friendship should be scaled up.

In Higham et al. (2007), the authors carry out tests on micro-array data and report that the generalised eigenvalue problem performs better at extracting information of biological interest. However, they focus on the extent to which the eigenvectors^7^ (i.e., the vectors {$\textbf{x}$} obtained by solving $\textbf{L}\textbf{x}=\lambda \textbf{D}\textbf{x}$) corresponding to the second and third smallest eigenvalues are able to reveal important features of the data by identifying relevant sub-clusters, whereas our interest lies in using the second smallest eigenvalue to infer how strongly connected a graph is. As will be shown in the “Comparison with ReHo” section, we have found that by upping the effect of weakly connected vertices, the generalised eigenvalue problem tends to be more sensitive to noise. With this in mind, we shall henceforth focus on the standard eigenvalue problem. This is also the default method employed in the latest version of the VB toolbox.

### The VB index as a modified cut-set weight

#### Relation of the algebraic connectivity to the ratio cut

A connected graph (so called because any two vertices are joined by a path consisting of one or more edges) may be disconnected by removing edges. Let us suppose that given a connected graph *G*, we do away with a collection of edges (called a *cut* or *edge cut*) and manage to divide *G* into **two** components, *B* and *C*. The corresponding *cut-set weight* can be obtained by summing the weights of the ‘cut’ edges:

20 $$\begin{aligned} \text {cut-set weight}(G)=\sum _{\begin{array}{c} v_i\in B\\ v_j\in C \end{array}}w_{ij}~~~(w_{ij}=0~\text {if}~e_{ij}\notin E). \end{aligned}$$

The rationale behind any clustering scheme is to group together vertices with strong affinity while separating those with divergent properties. In our case, the degree of similarity between any two vertices is reflected by the weight of the shared edge, and so we will attempt to partition the graph into two by removing the weakest edges. What we are interested in, therefore, is the cut that has the smallest weight.

To avoid instances when the cut-set weight is minimised by isolating a single vertex, we shall be using a modified cut-set weight (which we will henceforth refer to as the *ratio cut*)—one that takes into account the sizes of the resulting clusters (Von Luxburg 2007; Stanković et al. 2019; Wei and Cheng 1989):

21 $$\begin{aligned} \text {Ratio cut}(B,C) = \left( \frac{1}{n_B}+\frac{1}{n_C}\right) \sum _{\begin{array}{c} v_i\in B\\ v_j\in C \end{array}}w_{ij}. \end{aligned}$$

Here, $n_B$ ($n_C$) stands for the number of vertices in *B* (*C*).

The ratio cut can be expressed in terms of the graph Laplacian by means of Eq. (8). Let us consider a specific form for $\mathbf {{x}}$ (Stanković et al. 2019):

22 $$\begin{aligned} x_i={\left\{ \begin{array}{ll} 1/n_B~\text {if}~v_i\in B~;\\ -1/n_C~\text {if}~v_i\in C. \end{array}\right. } \end{aligned}$$

The magnitude (squared) of this vector is given by

23 $$\begin{aligned} \mathbf {{x}}^\text {T}\mathbf {{x}} = \left( \frac{1}{n_B}\right) ^2\times n_B +\left( -\frac{1}{n_C}\right) ^2\times n_C = \frac{1}{n_B}+\frac{1}{n_C}. \end{aligned}$$

Substituting for $x_i$ and $x_j$ in Eq. (8) yields (Stanković et al. 2019)

24 $$\begin{aligned} \mathbf {{x}}^\text {T}\textbf{L}\mathbf {{x}}&=\frac{1}{2}\sum _{\begin{array}{c} v_i\in B\\ v_j\in C \end{array}}w_{ij}\left[ \frac{1}{n_B}-\left( -\frac{1}{n_C}\right) \right] ^2 + \frac{1}{2}\sum _{\begin{array}{c} v_i\in C\\ v_j\in B \end{array}}w_{ij}\left( -\frac{1}{n_C}-\frac{1}{n_B}\right) ^2~; \end{aligned}$$

25 $$\begin{aligned}&=\frac{1}{2}\left( \frac{1}{n_B}+\frac{1}{n_C}\right) ^2\left( \sum _{\begin{array}{c} v_i\in B\\ v_j\in C \end{array}}w_{ij}+\sum _{\begin{array}{c} v_i\in C\\ v_j\in B \end{array}}w_{ij}\right) ~;\end{aligned}$$

26 $$\begin{aligned}&=\left( \frac{1}{n_B}+\frac{1}{n_C}\right) ^2\sum _{\begin{array}{c} v_i\in B\\ v_j\in C \end{array}}w_{ij}~~(\text {since the graph is undirected})~;\end{aligned}$$

27 $$\begin{aligned}&=\left( \frac{1}{n_B}+\frac{1}{n_C}\right) \times \text {Ratio cut}(B,C)\end{aligned}$$

[from Eq. (21)]

28 $$\begin{aligned}&=\mathbf {{x}}^\text {T}\mathbf {{x}}\times \text {Ratio cut}(B,C), \end{aligned}$$

and hence

29 $$\begin{aligned} \text {Min[Ratio cut($B$,$C$)]}=\text {Min}\left( \frac{\mathbf {{x}}^\text {T}\textbf{L}\mathbf {{x}}}{\mathbf {{x}}^\text {T}\mathbf {{x}}}\right) . \end{aligned}$$

We note, however, that this holds provided $\mathbf {{x}}$ is as specified in Eq. (22), which in turn implies that $\mathbf {{x}}$ must be orthogonal to $\mathbf {{1}}$, since (Stanković et al. 2019)

30 $$\begin{aligned} \mathbf {{x}}^\text {T}\mathbf {{1}}=\frac{1}{n_B}n_B+\left( -\frac{1}{n_C}\right) n_C=1-1=0. \end{aligned}$$

One common relaxation approach involves allowing the components of $\mathbf {{x}}$ to take arbitrary values in the set of real numbers. Therefore, we now endeavour to find a vector $\mathbf {{x}}\in {\mathbb {R}}^n$ that minimises the quantity $\mathbf {{x}}^\text {T}\textbf{L}\mathbf {{x}}/\mathbf {{x}}^\text {T}\mathbf {{x}}$ while still satisfying $\mathbf {{x}}^\text {T}\mathbf {{1}}=0$. As shown in the section “Minimising the Rayleigh quotient”, this vector is none other than the Fiedler vector. We may consequently combine Eqs. (14) and (29) into one relation:

31 $$\begin{aligned} \text {Min[Ratio cut}(B,C)]\approx \lambda _2, \end{aligned}$$

where the approximation sign reflects the fact that we have solved a relaxed version of Eq. (29).

In conclusion, we have shown that the second smallest eigenvalue of the graph Laplacian can be used to estimate a minimum value for the ratio cut, which is a sum of edge weights over all the edges removed to partition the graph into two clusters; this sum is weighted so that unbalanced clusters are penalized.

#### Scaling the algebraic connectivity

Our next goal is to define a scaled version of $\lambda _2$ that would be restricted to the range [0, 1]. It is well known that disconnected graphs have an algebraic connectivity of 0. At the other extreme, complete graphs (i.e., graphs in which every pair of vertices is connected via an edge) with maximally weighted edges have the largest value of $\lambda _2$, equal to the total number of vertices in the graph (so for a complete *n*-vertex graph whose edges all have a weight of 1, $\lambda _2=n$). We therefore scale the algebraic connectivity by the cardinality *n* of the vertex set (*n* is also called the *order* of the graph), and define the Vogt-Bailey (VB) index as follows:

32 $$\begin{aligned} \text {VB index} = \frac{\lambda _2}{n}. \end{aligned}$$

The difference between the scaling factor used here and the one in Bajada et al. (2020) stems from the fact that the original paper focuses on the generalised eigenvalue problem, $\textbf{D}^{-1}\textbf{L}\mathbf {{x}} = \lambda \mathbf {{x}}$, which in the case of a complete graph with maximally weighted edges returns a value for $\lambda _2$ equal to the mean of all eigenvalues except the smallest.

Substituting for $\lambda _2$ using Eqs. (21) and (31) yields

33 $$\begin{aligned} \text {VB index} \approx \frac{1}{n}\left( \frac{1}{n_{B_0}}+\frac{1}{n_{C_0}}\right) \sum _{\begin{array}{c} v_i\in B_0\\ v_j\in C_0 \end{array}}w_{ij} = \left( \frac{1}{n_{B_0}n_{C_0}}\right) \sum _{\begin{array}{c} v_i\in B_0\\ v_j\in C_0 \end{array}}w_{ij}, \end{aligned}$$

where a subscript 0 indicates that $B_0$ and $C_0$ are not just any two clusters, but the particular clusters that minimise the ratio cut, and the last equality was obtained by setting $n_{B_0}+n_{C_0}=n$. We shall henceforth refer to the cut-set weight $\sum _{v_i,v_j} w_{ij}~(v_i\in B_0,v_j\in C_0)$ as the *VB cut*.

As expressed in Eq. (33), the VB index is extremely intuitive: it is the summed weight of the edges removed, divided by the total number of these edges. We emphasise that the VB cut corresponds to a special cut—the one that minimises the ratio cut. Second, $n_{B_0}\times n_{C_0}$ amounts to the number of edges dispensed with only if each vertex in cluster $B_0$ is originally directly connected to every single vertex in $C_0$. This essentially means that graphs which are not complete to start with are reinterpreted as complete graphs having some edge weights equal to zero. In other words, the meaning of the VB index is best understood if we consider the number of edges detached from the graph to be fixed at $n_{B_0} \times n_{C_0}$, while the weights of those edges may vary—in the case of a complete graph with maximal weights, all edges have a weight of unity, and as a result, the VB index also equates to one, while a weakly connected graph has edges with smaller weights (and possibly some with a weight of zero, i.e., missing edges) and this lowers the VB index. It follows that a high VB index reflects the presence of ‘heavy’ edges and thus points to underlying voxels with strongly-correlated fMRI time series, while edges with small weights—due to weak correlations in said series—translate into a low VB value. Accordingly, a small VB index indicates a sharp change in local brain function.

## Results

### Testing the relation between the minimum ratio cut and the algebraic connectivity

To test how well the approximation of Eq. (31) holds, we assembled the following three sets of $27\times 27$ affinity matrices:

- Set 1: 1061 matrices constructed from the resting-state fMRI data for 2 participants [source: the Autism Brain Imaging Data Exchange (ABIDE I) Preprocessed data set (Craddock et al. 2013)].
- Set 2: 5074 matrices from the resting-state fMRI data for 10 participants [source: the minimally preprocessed Human Connectome Project (HCP) Young Adult data set (Van Essen et al. 2013; Glasser et al. 2013; Moeller et al. 2010; Feinberg et al. 2010; Setsompop et al. 2012; Xu et al. 2012; Jenkinson et al. 2002, 2012; Fischl 2012; Van Essen et al. 2011; Robinson et al. 2014, 2018)].
- Set 3: 118 matrices generated by sampling uniformly from the interval [0, 1].

**Fig. 1:**
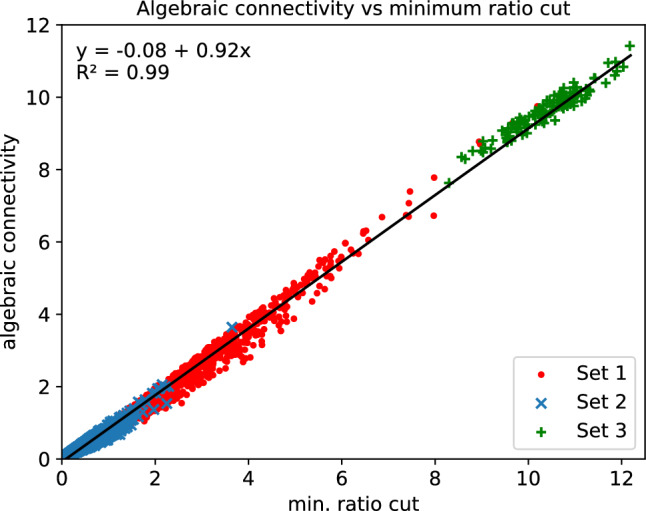
Algebraic connectivity vs minimum ratio cut for three sets of affinity matrices. The former is equivalent to $\lambda _2$, the second smallest eigenvalue of the graph Laplacian. The plot indicates that the minimum ratio cut is well approximated by the algebraic connectivity

Every matrix in Sets 1 and 2 corresponds to the graph of a randomly chosen vertex on the midthickness surface of the brain, and was calculated by applying Eqs. (3) and (4) to the fMRI data of a ‘neighbourhood’ consisting of the voxel containing the vertex and nearby voxels. For all 6253 matrices, we worked out $\lambda _2$ in the manner outlined at the end of the “Preliminaries” section and in Bajada et al. (2020),^8^ and also determined the minimum ratio cut. The latter was computed by means of an exhaustive search over all possible 2-cluster partitions. The results are displayed in Fig. 1, from which it is immediately apparent that the relation given by Eq. (31) provides a good fit to the data, and compares well with the equation obtained via linear least-squares regression ($y=0.92x-0.08$). The plot also reveals that matrices in Set 1 have, in general, a higher degree of connectivity than those in Set 2. This may be explained by the lower spatial resolution of the ABIDE data (3 mm isotropic, versus 2 mm isotropic for the HCP data). The way in which the matrices in Set 3 were built makes it difficult to find a ‘fault line’ in the associated graphs, and consequently, these graphs are harder to disconnect.

### Comparison with ReHo

ReHo is arguably the method most commonly used in the literature to study local homogeneities in brain function (Zang et al. 2004; Sun et al. 2018; Xu et al. 2019; Long et al. 2018; Yue et al. 2020; Zhu et al. 2020; Lin et al. 2015; Hu et al. 2022; Yang et al. 2018; Zuo et al. 2013). In this section, we compare its performance with that of the VB index, the aim being to understand how these two measures differ in what they can tell us about activity in the brain.

The **Re**gional **Ho**mogeneity approach (ReHo) (Zang et al. 2004) employs Kendall’s coefficient of concordance (*W*) to gauge the degree of synchronisation among the time series of a general voxel and those of its nearest neighbours. *W* is given by (Zang et al. 2004)

34 $$\begin{aligned} W = \frac{12R}{m^2(k^3-k)}, \end{aligned}$$

where *m* is the number of voxels in the neighbourhood, each with an associated time series of length *k*, and $R=\sum _{i=1}^k(R_i-\bar{R})^2$. The quantity $R_i$ is defined as the sum rank of the *i*th data point. It is calculated as follows: we rank the *k* data points making up the time series of a given voxel, and repeat for all voxels in the neighbourhood. Then, we sum the rankings of the *i*th data point across the *m* voxels. $\bar{R}$ is simply the mean sum rank: $\bar{R}=(\sum _{i=1}^k R_i)/k$. *W* takes a value in the range [0, 1], with 1 indicating perfect synchronisation among the time series—i.e., a situation in which the value of the signal at a given time point gets the same ranking for all voxels—and 0 denoting that the time series are completely out of sync. That said, a null score becomes highly improbable if *k* is not equal to *m*, whereas the VB index is always zero for a disconnected graph.

**Fig. 2:**
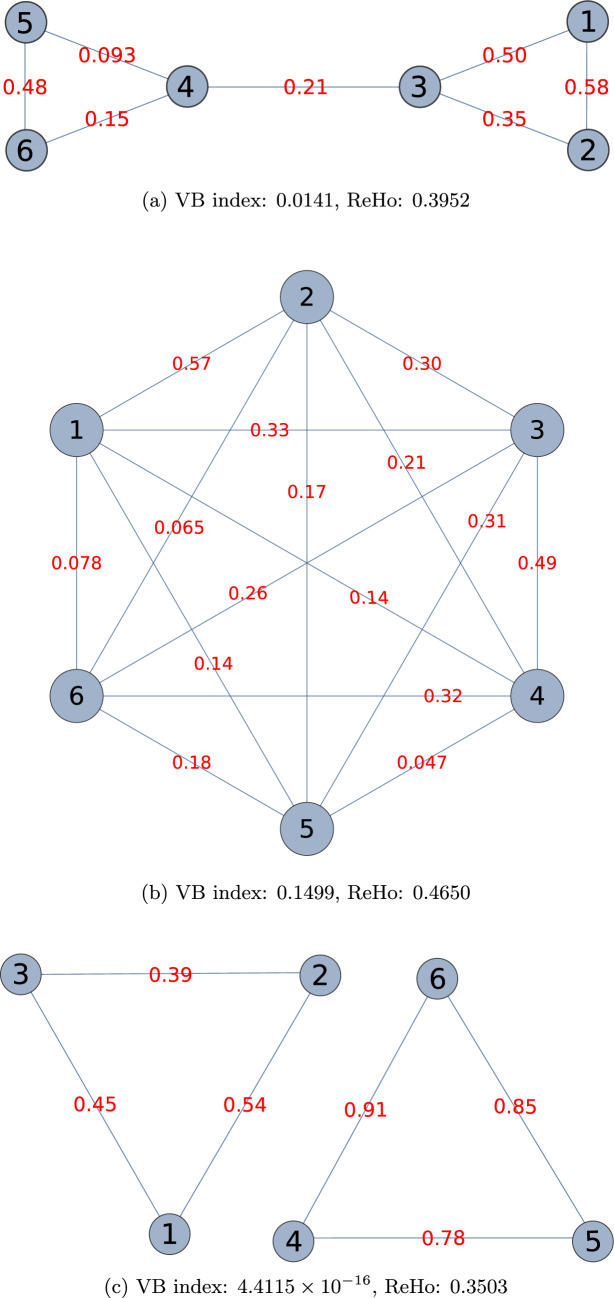
Different ways of joining 6 vertices into a graph. The examples shown are **a** sparsely connected, **b** fully connected (complete), and **c** disconnected

To compare the performance of the VB and ReHo homogeneity measures, we consider three examples: a sparsely connected graph (Fig. 2a), a complete graph (Fig. 2b), and a disconnected one (Fig. 2c). These graphs were constructed by assigning a time series^9^ of length 20 to each vertex and calculating the affinity matrix as detailed in the “Preliminaries” section. The entry $a_{ij}$ in the affinity matrix, derived from the correlation between the time series of vertices $v_i$ and $v_j$, then serves as the weight of the edge joining the two vertices. We generated the data in such a way that if the time series of a vertex is thought of as a vector, two vertices not directly linked by an edge would have orthogonal time series, which in turn would imply an edge with essentially zero weight. In the three cases represented in Fig. 2, the ReHo value turns out to be higher than the VB index. It is especially interesting to note that while the latter is approximately zero for the disconnected graph, and varies by an order of magnitude between the sparsely connected and complete graphs, the values we get with ReHo are comparable across all three examples.

Next, we tested ReHo and the VB index using synthetic fMRI data produced with the R software package neuRosim (Welvaert et al. 2011). The data had a signal-to-noise ratio of 3 and consisted of 5 spherical task activations with hard edges, superimposed on a mixture of the following noise components (Welvaert et al. 2011):

- *white*—a Rician distribution with non-centrality parameter of 0 (5%);
- *temporal*—an autoregressive model of order 3 (10%);
- *low frequency drift*—the frequency was set to 128 s (1%);
- *physiological*—noise due to heart beat and respiration (9%);
- *task*—noise due to spontaneous neural activity at the activation sites (5%);
- *spatial*—a Gaussian random field generated by a kernel having a full width at half maximum of 4 (70%).

**Fig. 3:**
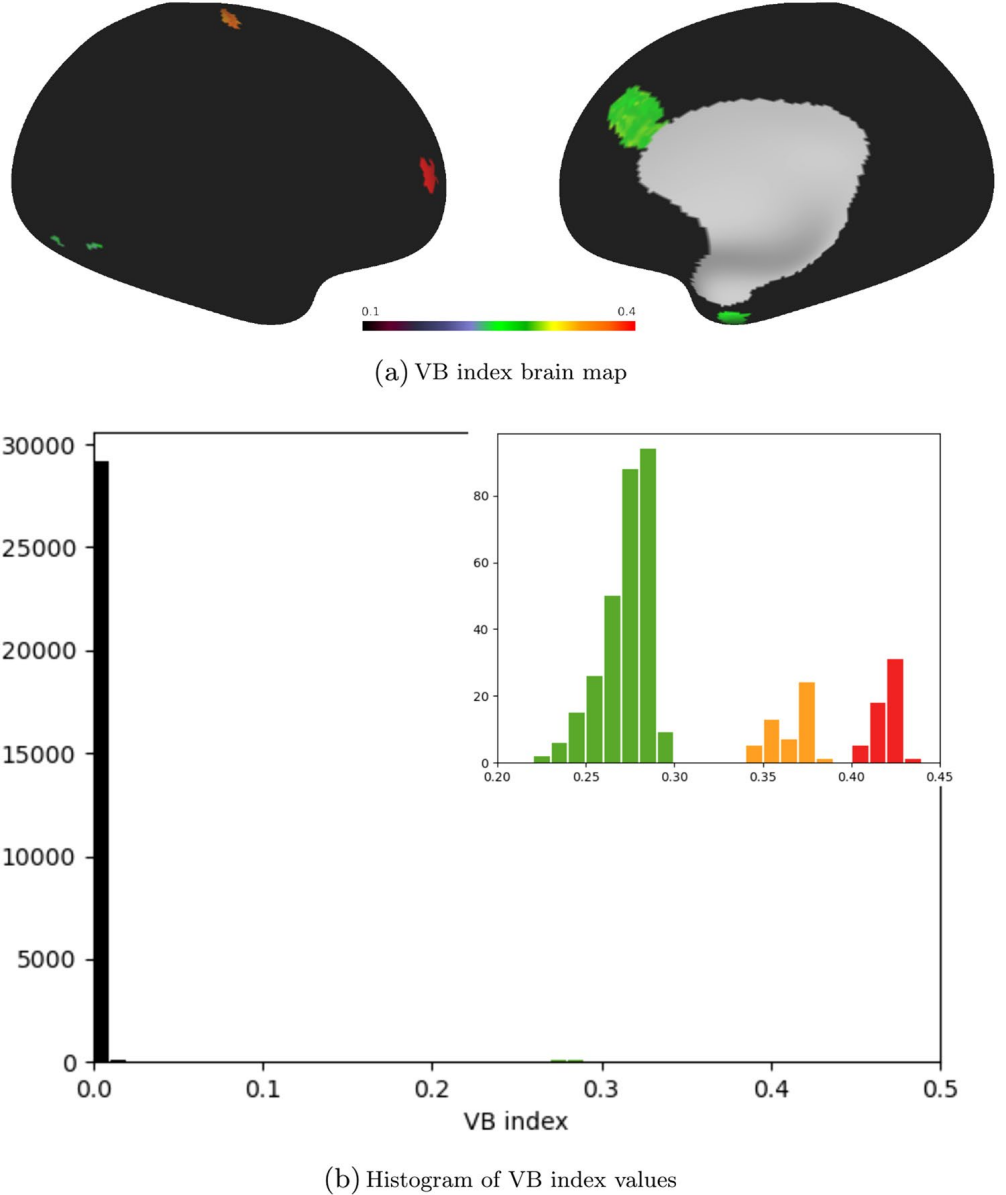
Brain map of VB index values (top) and associated histogram (bottom). The greater majority of the synthetic data provided to the algorithm represent noise and have a VB index close to zero. The inset shows 3 clusters at higher VB values. These clusters arise due to the task activations superimposed on the noise and have colour correspondence with the activated regions in the top panel

**Fig. 4:**
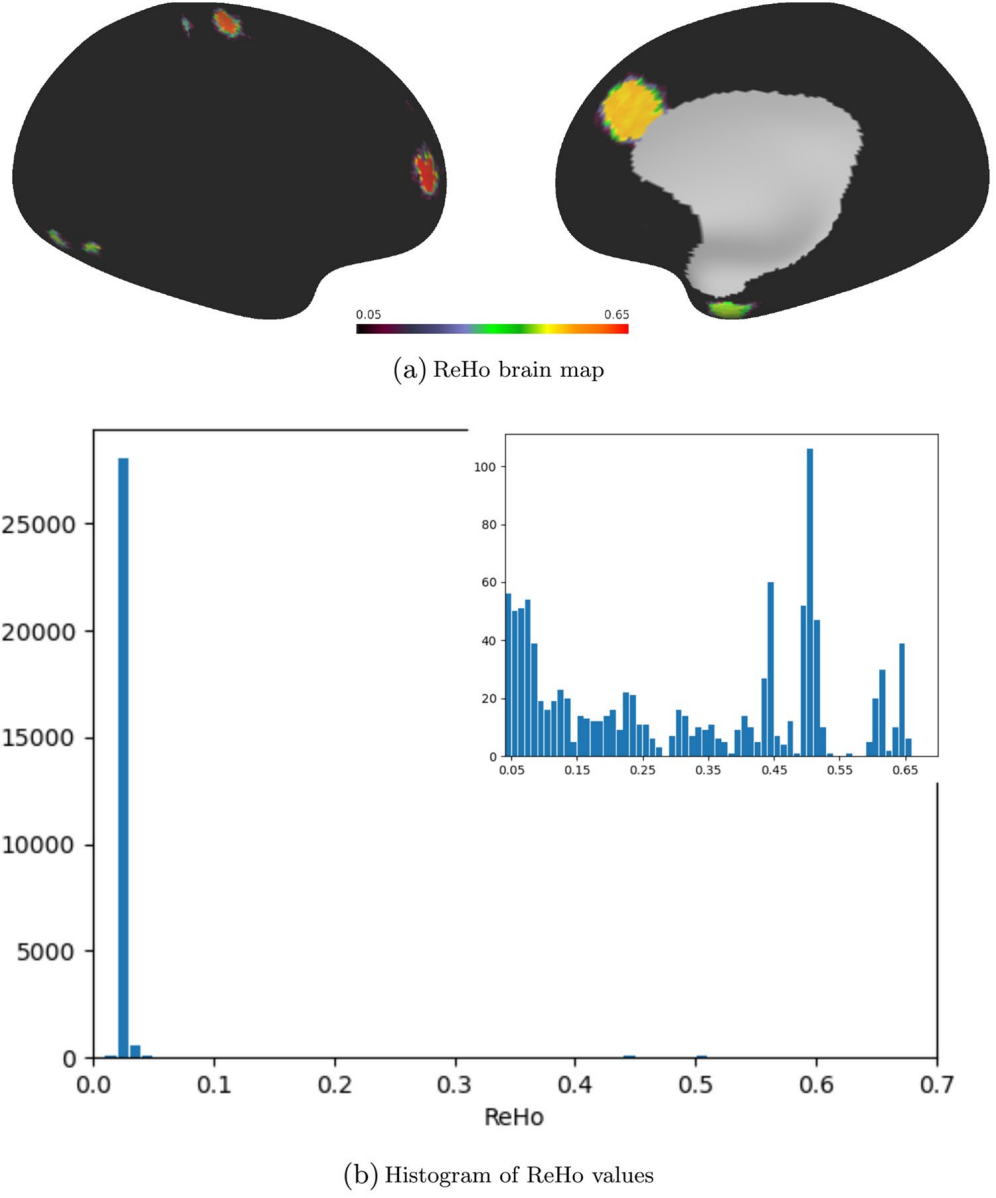
Brain map of ReHo values (top) and associated histogram (bottom; the inset displays data binned over a sub-interval). Like the VB index, the ReHo metric clearly differentiates between the activated regions and the background noise, but the distinction among the activations themselves is significantly sharper in the case of the VB index

**Fig. 5:**
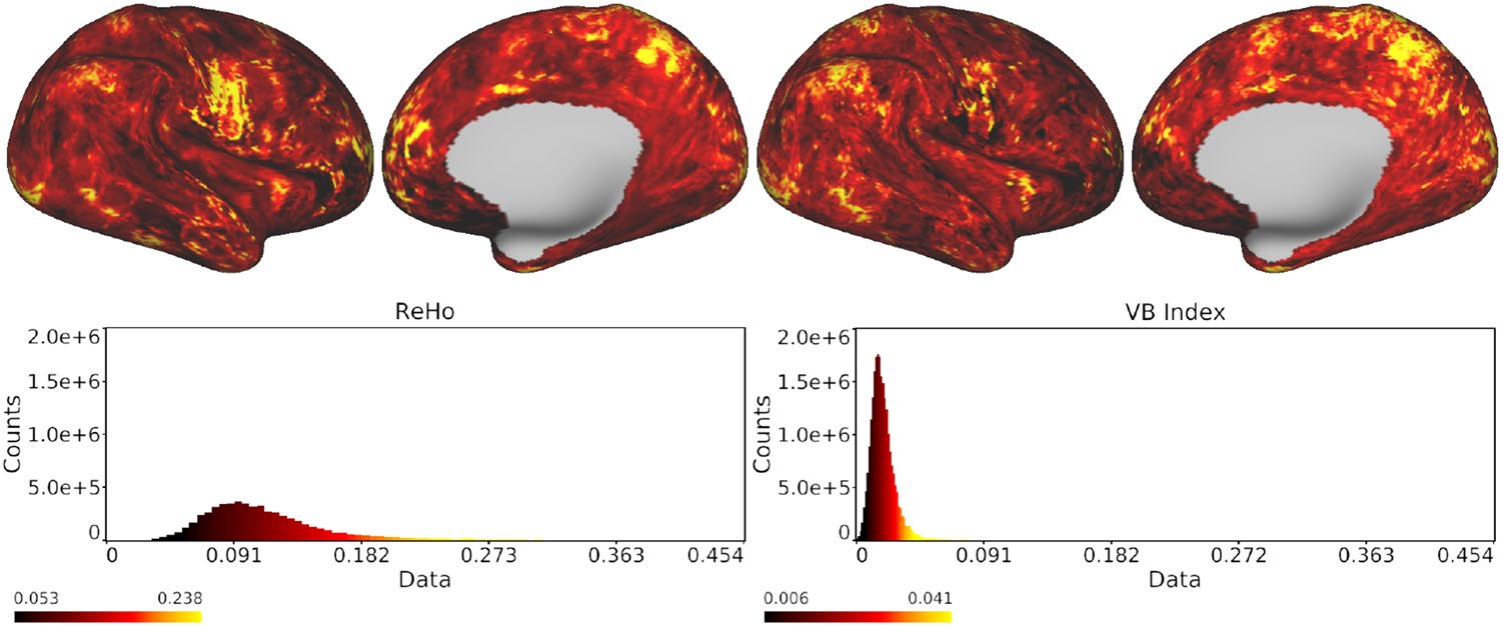
Local homogeneity of motor task data as estimated with the ReHo (left) and VB (right) algorithms. The histograms in the bottom panel show the distribution of values in the brain maps above. The reader is reminded, however, that local correlations of real data may contain artefacts arising from interpolation (Farrugia et al. 2023); consequently, this figure is provided for demonstrative purposes and should not be used to draw any inferences on motor tasks

**Fig. 6:**
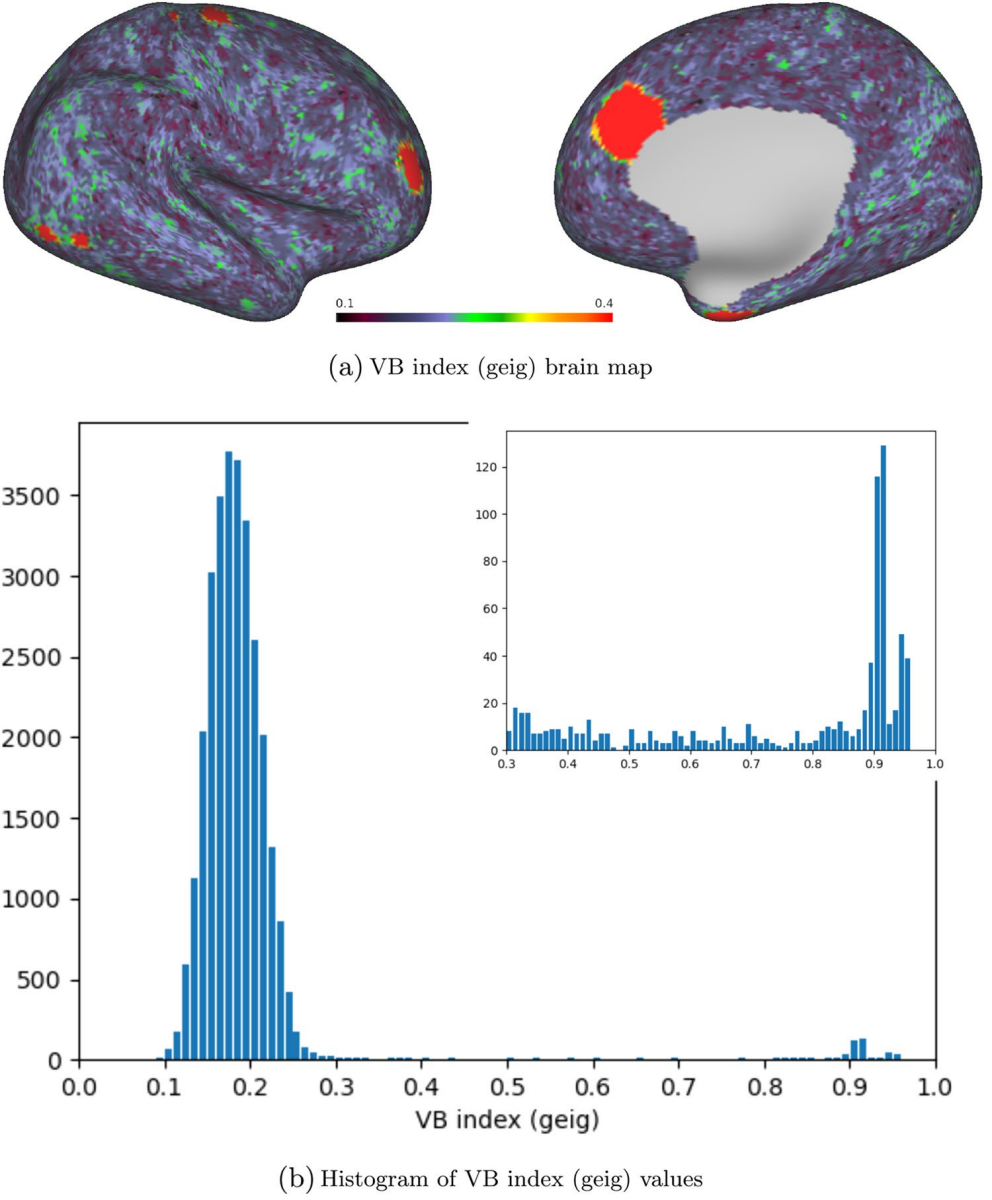
Brain map and histogram for the VB values obtained with the geig method. The inset presents the data binned over a sub-interval to the right of the principal peak

The synthetic data were processed by a hybrid algorithm which maps a given vertex on the midthickness surface of the brain to the corresponding voxel. It then calculates the ReHo or VB index value for a 27-voxel neighbourhood centred around (and including) the principal voxel, and maps the result back to the original vertex. The algorithm proceeds this way in a vertex-wise manner and at the end returns a VB or ReHo map for the entire brain surface. The resting-state data used to generate a baseline image and volumetric mask for the construction of synthetic time series (the volumetric mask is also required to run the version of the toolbox employed for this paper), as well as the midthickness surface and cortical mask provided as input to the hybrid algorithm, were obtained from the minimally preprocessed HCP Young Adult data set (Van Essen et al. 2013; Glasser et al. 2013; Moeller et al. 2010; Feinberg et al. 2010; Setsompop et al. 2012; Xu et al. 2012; Jenkinson et al. 2002, 2012; Fischl 2012; Van Essen et al. 2011; Robinson et al. 2014, 2018) by randomly selecting one participant.

The outputted VB and ReHo brain maps are presented in Figs. 3 and 4, respectively. It can be seen that both algorithms easily identify the activations, and as anticipated, produce an adequate level of contrast between them and the underlying noise. However, the activated regions have sharper edges in the VB map, and the accompanying histograms reflect this clearly. In the case of the VB index, the part of the histogram at the upper half of the range of VB values forms separate clusters that directly correspond to the areas of activation, but the histogram for the ReHo metric does not share this feature.

These results are corroborated when the VB and ReHo algorithms are tested on real data. The data in question consist of the HCP Young Adult Motor Task fMRI Preprocessed set (tfMRI_MOTOR_RL) (Van Essen et al. 2013; Glasser et al. 2013; Moeller et al. 2010; Feinberg et al. 2010; Setsompop et al. 2012; Xu et al. 2012; Jenkinson et al. 2002, 2012; Fischl 2012; Van Essen et al. 2011; Robinson et al. 2014, 2018) for the same participant selected when constructing the synthetic data (with midthickness and highly inflated surfaces and cortical masks taken from the Structural Preprocessed data for the participant). The brain maps obtained in this case are presented in Fig. 5 and show the same patterns of activation for both methods, which confirms the validity of the VB index as a measure of the local homogeneity of functional data. We again notice that ReHo tends to output higher values and introduces a degree of smoothness at the edges of activated regions with respect to the VB approach. This relative smoothness can be deduced by comparing the activations in the parietal lobe, for instance, and from the spread of the respective histograms. VB and ReHo brain maps for the motor task fMRI data of two other HCP subjects, and the corresponding histograms, are provided as supplementary material (Online Resource 1).

The synthetic data used previously were also fed into the VB toolbox with the Laplacian normalisation set to geig, which instructs the algorithm to return the eigenvalues of the generalised eigenvalue problem (Eq. 17). Figure 6 shows the distribution of VB index values obtained, both as a brain map and as a histogram. It is immediately apparent that the geig method distinguishes much less sharply among the individual activated regions than the original approach (unnorm), which, we recall, is based on the standard eigenvalue problem ($\textbf{L}\textbf{x}=\lambda \textbf{x}$). Additionally, the VB indices that geig outputs for the noise component have higher values and greater variance than their unnorm counterparts, indicating elevated  sensitivity to noise.

## Discussion and conclusion

The VB index is an ‘edge-detection algorithm’ introduced in Bajada et al. (2020) to look for sharp changes (‘edges’) in the local functional organization of the human cortex. In this work, we expound on the details of the underlying mathematical framework. In particular, we re-interpret the VB index as a modified cut-set weight associated with a particular graph cut—one that finds a trade-off between eliminating as few edges as possible and having clusters with a comparable number of vertices. This makes the VB index extremely intuitive. We test the approximation on which our interpretation is based using matrices extracted from real data, and conclude that it holds very well. Additionally, we introduce the concept of a VB cut (which is simply the sum of edge weights associated with the cut mentioned above), and show that the VB index can be understood as the VB cut divided by the total number of edges removed to partition the graph into two (provided missing edges are treated as edges with null weight).

Next, we compare the performance of the VB index with that of ReHo, a metric commonly used to assess regional homogeneity in brain function. The two algorithms can be executed with a similar amount of computational effort. We apply them to synthetic functional MRI data generated with the neuRosim package (Welvaert et al. 2011), and plot histograms for the output. While both ReHo and the VB index pick out the areas of activation from the background noise, the VB index traces sharper borders around these areas, localising the activations with greater precision. This might be due to an important distinction between the way ReHo and the VB index work—namely, while ReHo ranks data series, the VB index makes use of a modified version of the Pearson correlation coefficient. The ReHo metric is not dependent on the values of the data points per se, but rather on the way they are ordered (when ranked). So as an activation turns on and the signal starts to change, it is plausible that said change would not immediately be detectable in the ReHo output for the region. On the other hand, a variation in the values of the time series would still affect the VB index even if the ordering itself remains the same. Given the recent significant increase in the use of local homogeneity to probe functional anomalies associated with certain diseases and conditions—as elaborated on in the Introduction—we believe that the results outlined in this work are promising and the merits of the VB index warrant further investigation.

We also consider whether solving the generalised eigenvalue problem in place of the standard one to calculate the VB index has any benefits. Our results show that when determined this way, the VB index is more sensitive to noise and does not distinguish as well among the different regions of activation. Consequently, new versions of the VB toolbox will, by default, employ the regular Laplacian and the standard eigenvalue problem.

### Supplementary Information

Below is the link to the electronic supplementary material.

Supplementary file 1 (pdf 1901 KB)

## Data Availability

Data were provided in part by the Human Connectome Project, WU-Minn Consortium (Principal Investigators: David Van Essen and Kamil Ugurbil; 1U54MH091657) funded by the 16 NIH Institutes and Centers that support the NIH Blueprint for Neuroscience Research; and by the McDonnell Center for Systems Neuroscience at Washington University. Data from the ABIDE I (Autism Brain Imaging Data Exchange) Preprocessed data set were also used. The matrices produced for Fig. 1 may be retrieved from https://doi.org/10.5281/zenodo.8246344 (Farrugia 2023b). The scene file for Figs. 3, 4 and 6 is available at https://balsa.wustl.edu/study/jN6Xl (Farrugia 2023c), together with the synthetic fMRI data and the volumetric mask required as input to the VB toolbox.

## Footnotes

1 The larger the number of edges and the weights assigned to them, the more strongly connected the graph is, and the greater the challenge to break it up into separate parts.

2 As used in this article, ‘searchlight’ refers to the fact that the VB index is computed over a small neighbourhood surrounding each vertex on the midthickness surface of the brain. The vertex is mapped to the voxel that contains it, and the neighbourhood is constructed as a 27-voxel cube (provided all 27 voxels lie within the brain), with the original voxel at its centre. Importantly, however, the methodology can accommodate any definition of neighbourhood.

3 The proof is provided with the supplementary data of the companion paper (Bajada et al. 2020). A superscript T indicates that a quantity is transposed.

4 This constraint may be relaxed in the manner of Higham et al. (2007).

5 Since $\textbf{L}$ is a symmetric matrix, it has a full set of linearly independent (mutually orthogonal) eigenvectors.

6 The equation $\textbf{L}\textbf{x} = \lambda \textbf{D}\textbf{x}$ may also be cast in the form $\textbf{L}_\text {SYM}^{}\mathbf {{y}} = \lambda \mathbf {{y}}$, where $\textbf{L}_\text {SYM}^{}$, the *symmetric normalised Laplacian*, is defined as $\textbf{D}^{-1/2}\textbf{L} \textbf{D}^{-1/2}$, and $\textbf{y}=\textbf{D}^{1/2}\,\textbf{x}$ (Bajada et al. 2020) (equivalently, $\textbf{x}=\textbf{D}^{-1/2}\,\textbf{y}$). In this case, the edges would remain undirected. Note that $\textbf{D}^{-1/2}$ can be determined from $\textbf{D}$ by finding the reciprocal of the elements along the main diagonal of $\textbf{D}$ and computing their square root.

7 Strictly speaking, the authors work with the symmetric normalised Laplacian and adopt $\textbf{D}^{-1/2}\,\textbf{y}_2$ as the normalised Fiedler vector ($\textbf{x}$ and $\textbf{y}$ have the same meaning as in Footnote 6, and $\textbf{y}_2$ is the eigenvector corresponding to the second smallest eigenvalue of $\textbf{L}_\text {SYM}^{}$).

8 Note, however, that the method of Bajada et al. (2020) is surface-based, whereas we employ the hybrid algorithm mentioned in the Introduction.

9 The time series were constructed by sampling at regular intervals from a sine wave with a noise component drawn randomly from a normal distribution. Distinct series differed in the initial phase of the sine function, as well as in the mean and variance of the normal distribution.

## Code availability

The version of the VB toolbox employed in this work may be obtained from GitHub (https://github.com/VBIndex/py_vb_toolbox/tree/Local-gradients-paper). Installation instructions are provided in the README file. Kindly note that the Laplacian normalisation method still defaults to geig in this version.

The brain maps in Figs. 3–6 were prepared with the Connectome Workbench software, v1.4.2 (Marcus et al. 2011), while the graphs in Fig. 2 were generated with Wolfram Mathematica v13.0.1.0 (Wolfram Research, Inc. Illinois, 2021). The code for Fig. 2(a) may be found at https://notebookarchive.org/2023-05-4m1mlah (Farrugia 2023a) and can easily be adapted to other graph structures, including those of Figs. 2(b) and 2(c).
