# Supplementary material for: Local gradient analysis of human brain function using the Vogt-Bailey Index
Source: Brain Struct Funct. 2024 Jan 31;229(2):497–512. doi: 10.1007/s00429-023-02751-7 (PMC10917869; doi:10.1007/s00429-023-02751-7)
Supplement: Supplementary file 1 — (pdf 1901 KB) [file 429_2023_2751_MOESM1_ESM.pdf]

Local gradient analysis of human brain function using the Vogt-Bailey Index

Brain Structure and Function

Christine Farrugia*[1,2,3], Paola Galdi[4], Irati Arenzana Irazu[5], Kenneth Scerri[1], and Claude J. Bajada†[6,2]

[1]Faculty of Engineering, L-Università ta' Malta, Msida, Malta
[2]University of Malta Magnetic Resonance Imaging Platform (UMRI), L-Università ta' Malta, Msida, Malta
[3]Centre for Clinical Brain Sciences, The University of Edinburgh, Edinburgh, UK
[4]School of Informatics, The University of Edinburgh, Edinburgh, UK
[5]Faculty of Engineering, Mondragon Unibertsitatea, Mondragón, Spain
[6]Faculty of Medicine and Surgery, L-Università ta' Malta, Msida, Malta

The figures presented here were obtained by applying the VB and ReHo algorithms to the data of 2 subjects from the HCP Young Adult Motor Task fMRI Prepocessed set (tfMRI_MOTOR_LR) (Van Essen, Smith et al, 2013; Glasser et al, 2013; Moeller et al, 2010; Feinberg et al, 2010; Setsompop et al, 2012; Xu et al, 2012; Jenkinson, Bannister et al, 2002; Jenkinson, Beckmann et al, 2012; Fischl, 2012; Van Essen, Glasser et al, 2011; Robinson, Jbabdi et al, 2014; Robinson, Garcia et al, 2018). The midthickness and highly-inflated surfaces, as well as the cortical masks, were taken from the Structural Preprocessed data for the participants.

Top panel: Brain maps produced with the ReHo (left) and VB (right) methods.
Bottom panel: Histograms showing the distribution of values in the brain maps.

The reader is reminded that local correlations of real data may contain artefacts arising from interpolation (Farrugia et al, 2023); consequently, these figures are provided for demonstrative purposes and should not be used to draw any inferences on motor tasks.

*christine.farrugia@ed.ac.uk
†claude.bajada@um.edu.mt

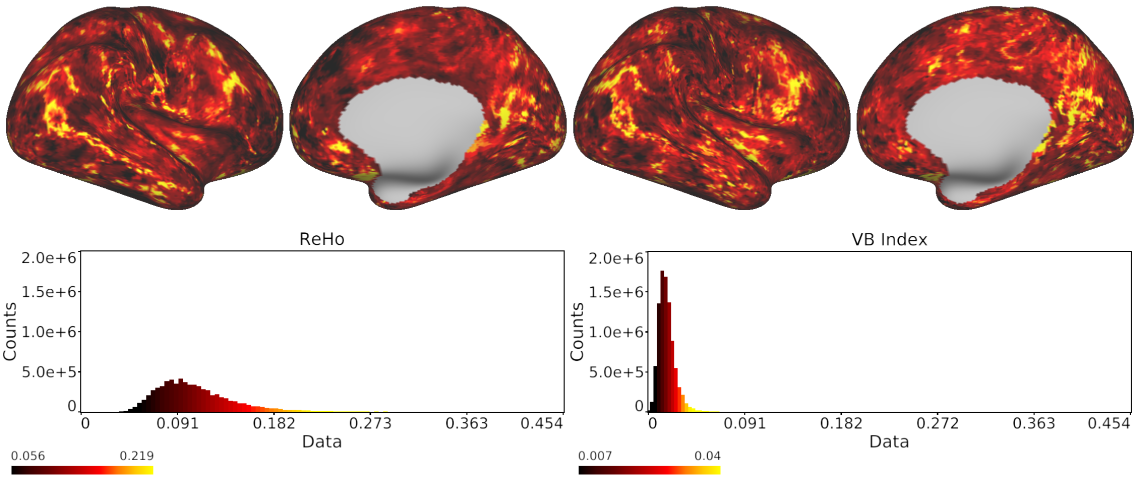

Fig. S1

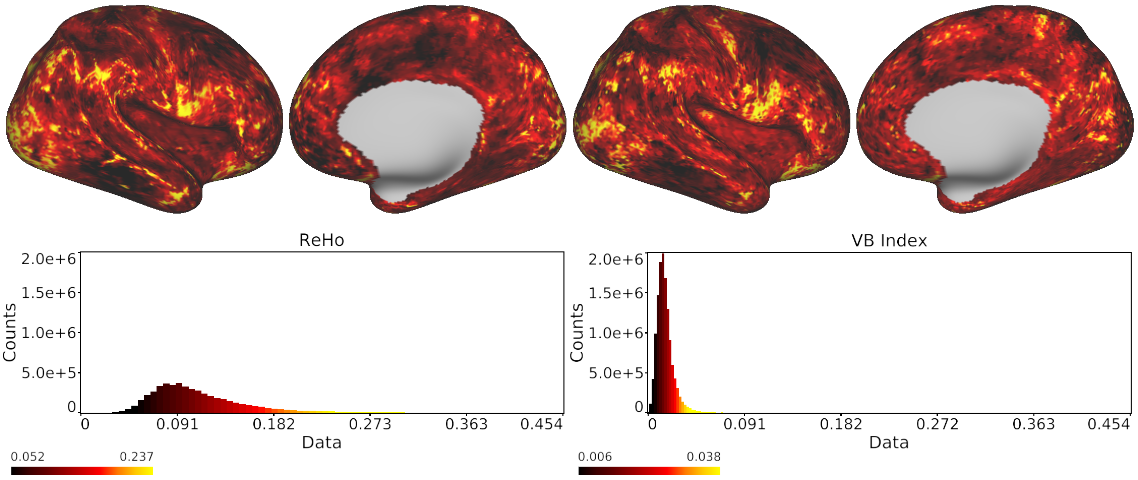

Fig. S2

References

Farrugia C, Smith RE, Bajada CJ (2023). Effects of preprocessing on local homogeneity of fMRI data. Poster abstract: the Organization for Human Brain Mapping annual meeting, Montréal. https://www.um.edu.mt/library/oar/handle/123456789/110643

Feinberg DA, Moeller S, Smith SM et al (2010). Multiplexed echo planar imaging for sub-second whole brain fMRI and fast diffusion imaging. PLoS ONE 5 (12):e15710. https://doi.org/10.1371/journal.pone.0015710

Fischl B (2012). FreeSurfer. NeuroImage 62:774. https://doi.org/10.1016/j.neuroimage.2012.01.021

Glasser MF, Sotiropoulos SN, Wilson JA et al (2013). The minimal preprocessing pipelines for the Human Connectome Project. NeuroImage 80:105. https://doi.org/10.1016/j.neuroimage.2013.04.127

Jenkinson M, Beckmann CF, Behrens TE, Woolrich MW, Smith SM (2012). FSL. NeuroImage 62:782. https://doi.org/10.1016/j.neuroimage.2011.09.015

Jenkinson M, Bannister P, Brady M, Smith S (2002). Improved optimization for the robust and accurate linear registration and motion correction of brain images. NeuroImage 17 (2):825. https://doi.org/10.1006/nimg.2002.1132

Moeller S, Yacoub E, Olman CA et al (2010). Multiband multislice GE-EPI at 7 tesla, with 16-fold acceleration using partial parallel imaging with application to high spatial and temporal whole-brain fMRI. Magn. Reson. Med. 63 (5):1144. https://doi.org/10.1002/mrm.22361

Robinson EC, Garcia K, Glasser MF et al (2018). Multimodal surface matching with higher-order smoothness constraints. NeuroImage 167:453. https://doi.org/10.1016/j.neuroimage.2017.10.037

Robinson EC, Jbabdi S, Glasser MF et al (2014). MSM: a new flexible framework for multimodal surface matching. NeuroImage 100:414. https://doi.org/10.1016/j.neuroimage.2014.05.069

Setsompop K, Gagoski BA, Polimeni JR et al (2012). Blipped-controlled aliasing in parallel imaging for simultaneous multislice echo planar imaging with reduced *g*-factor penalty. Magn. Reson. Med. 67 (5):1210. https://doi.org/10.1002/mrm.23097

Van Essen DC, Smith SM, Barch DM et al (2013). The WU-Minn Human Connectome Project: An overview. NeuroImage 80:62. https://doi.org/10.1016/j.neuroimage.2013.05.041

Van Essen DC, Glasser MF, Dierker DL, Harwell J, Coalson T (2011). Parcellations and hemispheric asymmetries of human cerebral cortex analyzed on surface-based atlases. Cereb. Cortex 22 (10):2241. https://doi.org/10.1093/cercor/bhr291

Xu J, Moeller S, Strupp J et al (2012). Highly accelerated whole brain imaging using aligned–blipped–controlled–aliasing multiband EPI. Proc. Int. Soc. Mag. Reson. Med. 20:2306
